# Enhancement of Aggregation-Induced Emission by Introducing Multiple *o*-Carborane Substitutions into Triphenylamine

**DOI:** 10.3390/molecules22112009

**Published:** 2017-11-19

**Authors:** Kenta Nishino, Kyoya Uemura, Masayuki Gon, Kazuo Tanaka, Yoshiki Chujo

**Affiliations:** Department of Polymer Chemistry, Graduate School of Engineering, Kyoto University, Katsura, Nishikyo-ku, Kyoto 615-8510, Japan; polyarco.24.k@gmail.com (K.N.); uemura@poly.synchem.kyoto-u.ac.jp (K.U.); gon@poly.synchem.kyoto-u.ac.jp (M.G.)

**Keywords:** carborane, twisted intramolecular charge transfer, triphenylamine

## Abstract

The enhancement of aggregation-induced emission (AIE) is presented on the basis of the strategy for improving solid-state luminescence by employing multiple *o*-carborane substituents. We synthesized the modified triphenylamines with various numbers of *o*-carborane units and compared their optical properties. From the optical measurements, the emission bands from the twisted intramolecular charge transfer (TICT) state were obtained from the modified triphenylamines. It was notable that emission efficiencies of the multi-substituted triphenylamines including two or three *o*-carborane units were enhanced 6- to 8-fold compared to those of the mono-substituted triphenylamine. According to mechanistic studies, it was proposed that the single *o*-carborane substitution can load the AIE property via the TICT mechanism. It was revealed that the additional *o*-carborane units contribute to improving solid-state emission by suppressing aggregation-caused quenching (ACQ). Subsequently, intense AIEs were obtained. This paper presents a new role of the *o*-carborane substituent in the enhancement of AIEs.

## 1. Introduction

Organic luminescent materials are versatile as a platform for developing not only opto-electronic devices but also optical bioprobes and sensors. By modulating chemical structures according to preprogrammed designs, material functions can be readily tuned. There are numerous numbers of luminescent organic molecules in the diluted solution, while the limited number of solid-state emissive dyes has been available, as most of luminescent properties are spoiled in the condensed state via aggregation-caused quenching (ACQ). One of the valid strategies for obtaining solid-state luminescent properties is to design materials by employing aggregation-induced emission (AIE)-inducible “element-blocks” [1], which are a minimum functional unit containing heteroatoms. It is known that AIE-active molecules and materials can provide intense emission only in the aggregation state, and because of a large versatility of AIE, a wide variety of applications have been achieved [2]. Recently, various types of AIE-inducible element-blocks have been discovered, and solid-state luminescent materials can be produced [3]. Additionally, luminescent chromic behaviors toward various external stimuli were occasionally observed from these AIE-active “element-block materials” [4]. Thus, exploration and comprehension of new AIE-active element-blocks are a topic with high relevance, particularly in material science.

*o*-Carborane [5,6,7,8,9,10] is an icosahedral cluster composed of 2 carbon and 10 boron atoms, and it has recently attracted attention as a key component for constructing solid-state luminescent materials [11,12,13,14,15,16,17,18,19,20,21,22]. Because of the electron-deficient nature of the boron cluster, intense emission from the intramolecular charge transfer (ICT) state can be observed from the donor–acceptor system with electron-donating units such as triphenylamine and its structural analogues [23,24,25,26]. In particular, owing to steric hindrance of the sphere shape of the cluster, suppression of ACQ was often induced in the condensed state, followed by intense solid-state emission [11,12,13,14,15,16,17,18,19,20,21,22,27,28,29]. Indeed, applicability of the modified *o*-carboranes for organic light-emitting devices has been demonstrated from the recent studies [30,31]. Moreover, it should be noted that some of the modified *o*-carboranes also showed AIE [23,24,30,32,33,34,35,36,37,38]. In the solution state, the modified *o*-carboranes presented slight emission as a result of emission annihilation by molecular motions, while intense emission was recovered because of low mobility in the solid state. Thus, we have also focused on *o*-carborane as an AIE-inducible element-block [3,39,40,41,42]. However, in the aggregation state, the AIE-active *o*-carboranes still suffer from a decrease in emission efficiency as a result of incomplete suppression of molecular motions in the aggregation state. Thus, our next goal is to establish design strategies for enhancing AIE.

Herein, we report the synthesis and optical properties of the modified triphenylamines with various numbers of *o*-carborane units. According to the recent works, it was observed that the aryl-modified *o*-carboranes provided extremely intense luminescence with almost quantitative efficiencies, even in the solid state [27,28,29]. Moreover, it was shown that multi-carborane substitutions onto benzene induced AIE and solid-state luminescence [43]. On the basis of these results, we presumed that the introduction of multiple *o*-carborane units into the strong electron-donating unit might be effective for realizing intense AIE. To examine the validity of this idea, we focused on triphenylamine as an electron-donor [23,24,25,26,30,31,32,44,45,46,47], synthesized modified triphenylamines with various numbers (1–3) of *o*-carboranes and compared their optical properties.

## 2. Results and Discussion

**Figure 1:**
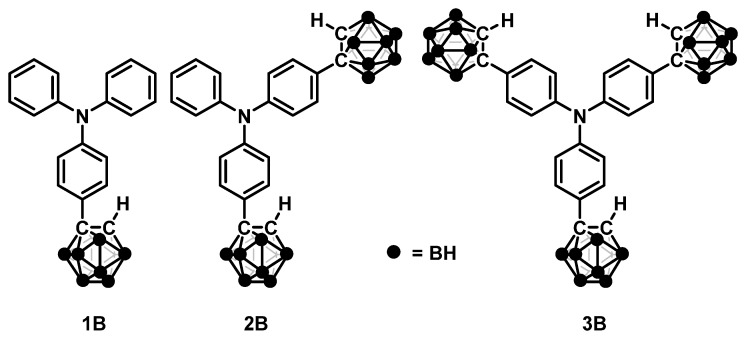
Chemical structures of the modified triphenylamines used in this study.

**Scheme 1:**
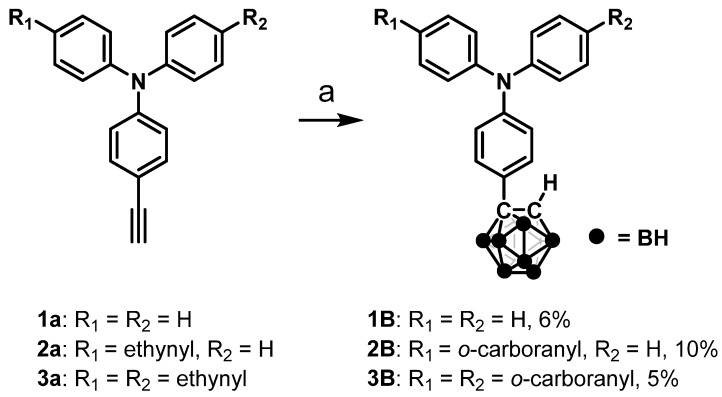
Syntheses of **1**–**3B** ^a^. (^a^ Reagents and condition: decaborane, AgNO_3_, toluene, and acetonitrile; reflux, 2 days for **1B** and 7 days for **2B** and **3B**.).

Modified triphenylamines with various numbers of *o*-carborane units were synthesized (Figure 1 and Scheme 1) [48]. The desired products were obtained via insertion of decaborane(14) to ethynyl compounds. All the products were characterized by ^1^H-, ^11^B- and ^13^C-NMR spectra and high-resolution mass measurements (Charts S1–S9). The products showed a high stability and good solubility in common organic solvents such as hexane, octane, benzene, dichloromethane, and tetrahydrofuran. In particular, the products provided strong luminescence in the solid state. Thus, further measurements were performed with the products.

**Figure 2:**
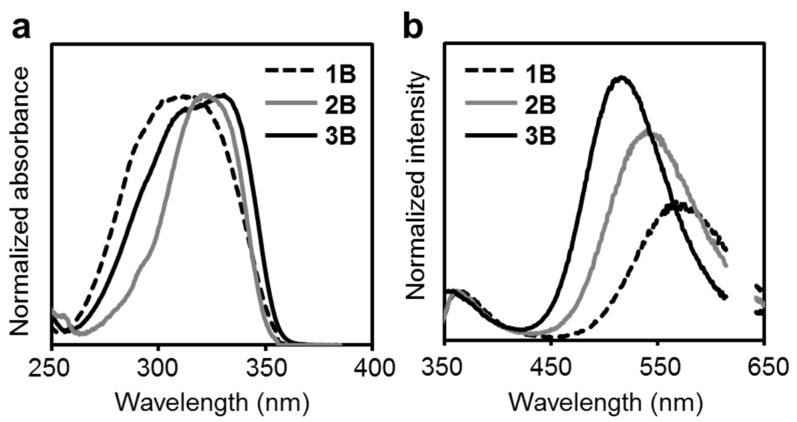
(**a**) UV–vis absorption and (**b**) photoluminescence PL spectra of the modified triphenylamines in octane (1.0 × 10^−5^ M). Light scattering from the incident light around 620 nm was omitted in the spectra.

Electronic structures of the modified triphenylamines in the ground state were evaluated with UV–vis absorption measurements (Figure 2a and Table 1). The spectra were obtained in the octane solution. All the modified triphenylamines showed almost the same absorption spectra, with peaks at around 320 nm. In addition, molar extinction coefficients at the absorption maxima were enhanced by increasing the number of *o*-carborane units. An increase of the number of chromophore units composing the *o*-carborane–aniline structure should be responsible for enhancing absorption properties.

Emission properties were examined with the modified triphenylamines. Triphenylamine scarcely presented photoluminescence (PL) [46,47], while all the modified triphenylamines showed two emission peaks at around 370 and 550 nm in the octane solution state, although the emission efficiencies were low (Figure 2b and Table 1 and Table S1). It was suggested that these emission bands in the near-UV and longer-wavelength regions were attributable to the locally excited (LE) and ICT states, respectively, because only the emission bands in the longer-wavelength region were shifted by changing the solvent polarity (Figure S1 and Table S2) [23,24,25,26].

Another significant point was the blue-shift of the emission peak in the longer-wavelength region by increasing the number of the *o*-carborane units. In the previous reports on electronic properties of the donor–acceptor system including triphenylamine, similar blue-shifts of absorption and emission bands have been observed by increasing the number of electron-accepting branches [49,50,51]. Accordingly, it is implied that electron-donating ability from lone pairs of nitrogen in the center of the triphenylamine moiety should be weakened by increasing the number of electron-deficient *o*-carborane substituents. Thereby, the blue-shift was induced in the emission band. Indeed, from the Lippert–Mataga plots and following analyses, it was indicated that the ICT characters were confirmed with similar extents to the previous donor–π–acceptor system [52]. In particular, it was noted that the degree of the ICT character decreased by the introduction of *o*-carborane (Figure S2 and Table S3). The differences in dipole moments during the excitation were extended by reducing the number of *o*-carborane units (**2B**: 14.0 D; **3B**: 13.5 D). These results correspond to the results from the previous reports and support the above mechanism.

**Figure 3:**
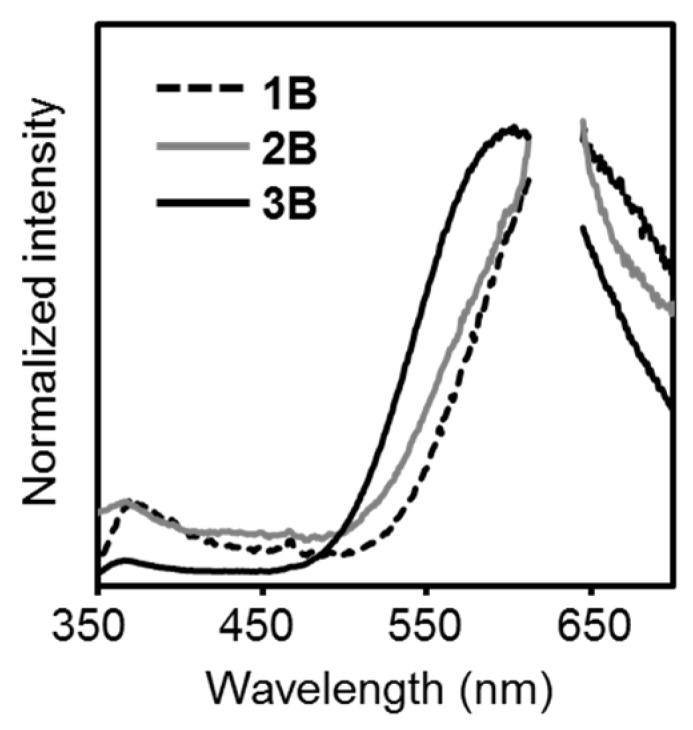
Photoluminescence (PL) spectra of (a) **1B**, (b) **2B** and (c) **3B** in the solid state at room temperature. Light scattering from the incident light at around 620 nm was omitted in the spectra.

Next, the solid-state luminescent properties were evaluated (Figure 3). Intense emission bands attributable to the ICT emission were obtained in the PL spectra with the solid samples. Significant enhancements of emission efficiencies were observed, comparing to those of the solution samples (Table 1). These results mean that the triphenylamines should be the AIE-active molecules. It should be noted that emission efficiencies of **2B** and **3B** were 6- to 8-fold greater than that of **1B**, clearly indicating that the introduction of multiple *o*-carborane substituents should be valid for enhancing AIE. Owing to steric hindrances of the multiple *o*-carborane units, ACQ could be synergistically suppressed even in the solid state. Thus, significant intense emission bands could be observed from the solid samples of the multi-substituted triphenylamines. By cooling the samples at 77 K, the intensity of the ICT emission bands increased (Figure S3). Thermal motions should be inhibited, and emission enhancement was observed.

**Figure 4:**
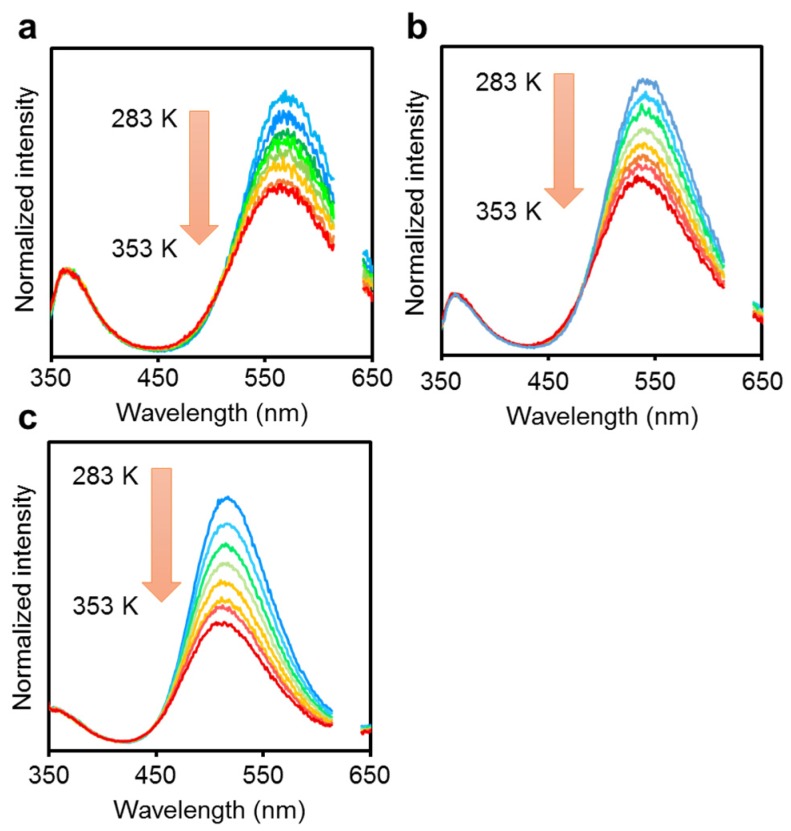
Variable temperature photoluminescence (PL) spectra of (a) **1B**, (b) **2B** and (c) **3B** in octane (1.0 × 10^−5^ M) from 283 to 353 K. Light scattering from the incident light at around 620 nm was omitted in the spectra.

To clarify the emission mechanism, further analyses were executed. According to the previous work with the anthracene-modified *o*-carborane dyad, it was shown that the ICT emission is obtained via the twisted intramolecular charge transfer (TICT) mechanism in the crystalline state [53]. The temperature dependency of the dual emission properties would provide mechanistic information. In addition, the dual emission properties are useful for realizing luminescent chromic materials toward environmental changes such as temperature, although emission efficiencies in the solution were small [54]. Therefore, we measured the PL spectra at various temperatures in the octane solution (Figure 4). Thermochromic luminescent behaviors were observed from all the solutions. The intensity ratio of the emission band in the longer-wavelength region decreased by increasing the solution temperature, implying that this emission band was from the TICT state in which structural alteration in the excited state occurred [53]. The rotation barrier (*E*_a_) and energy gaps between the LE state and ICT states (Δ*H*) can be estimated from Stevens–Ban plots based on variable-temperature PL spectra presenting dual emission (Figure S4). Accordingly, almost identical values were obtained from all the modified triphenylamines (Table 1). These data suggest that conformation changes should proceed independently from the substitution effect of the *o*-carborane unit after excitation.

PL spectra in 2-methyl-THF (MTHF) solution at 77 K were recorded to understand its conformations in the ground state (Figure S5). Three emission bands with peaks at around 360, 450 and 640 nm were observed from all the modified triphenylamines. Emission peaks at around 360 and 640 nm were assigned to the transitions from the LE and ICT states, respectively. The emission band at around 450 nm showed a long decay time (4.2 ms) and corresponded to phosphorescence of triphenylamine (Figure S6). Thus, this emission band was attributable to phosphorescence from the triphenylamine moiety.

**Figure 5:**
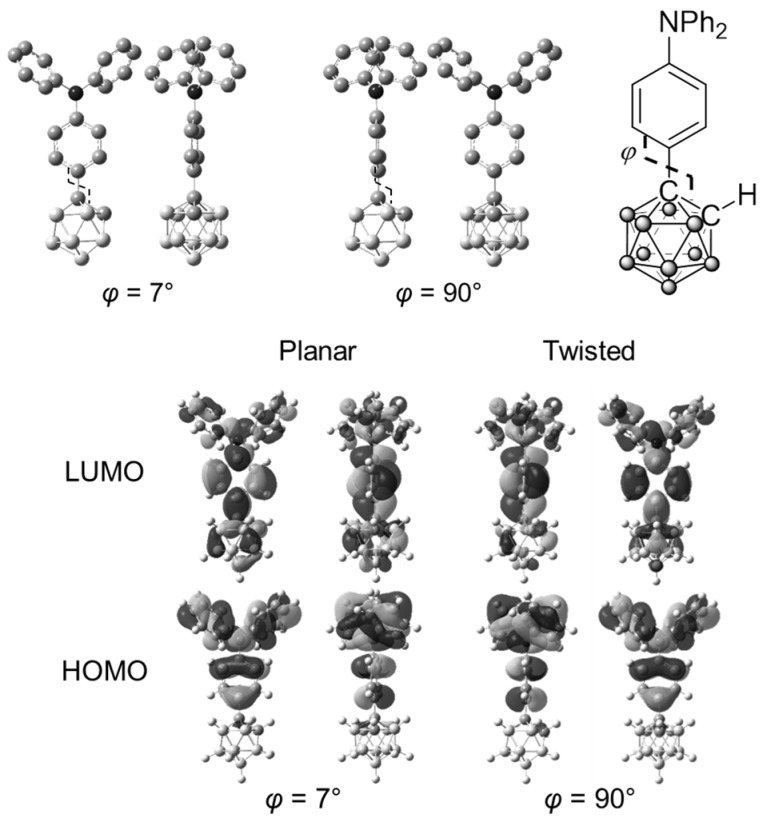
Optimized structures and calculated molecular orbitals of **1B** in the ground state.

**Figure 6:**
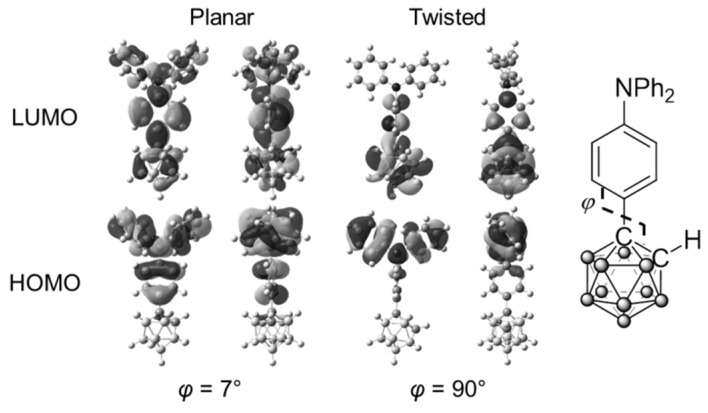
Calculated molecular orbitals in the transitions of **1B** in the excited state.

According to the previous reports, it is known that electronic structures of *o*-carborane are drastically changed by the rotation at the substituents [55]. To obtain deep insight into the optical properties, theoretical calculations were performed with **1B** (Figure 5 and Figure 6). The optimized structures and frontier orbitals (HOMO: highest occupied molecular orbital; LUMO: lowest unoccupied molecular orbital) were calculated by using density functional theory (DFT) and time-dependent DFT (TD-DFT) at the CAM-B3LYP/6-31+G(d,p)//B3LYP/6-31G(d) level by using the Gaussian09 program in the ground and excited states, respectively. The dihedral angle between the C–C bonds in *o*-carborane and the hypothetical plane of triphenylamine was denoted as *φ*. It was shown that two conformations (*φ* = 7° and 90°) with similar total energy levels should exist in the ground state of **1B** (Figure 5). In the excited state, HOMO was located at the triphenylamine moiety, and LUMO was delocalized through the whole molecule in the planar conformation (Figure 6). In contrast, in the twisted conformation, LUMO was localized at the *o*-carborane unit, although a similar orbital distribution of HOMO to that in the planar conformation was obtained. This result corresponds to those from the previous aryl-modified *o*-carborane dyads, which can present the TICT emission [53]. In the planar conformation (*φ* = 7°), electronic interaction scarcely occurred between the *o*-carborane unit and the triphenylamine moiety. As a result, mainly the LE emission could be obtained. On the other hand, the ICT emission was induced in the twisted state. In the solution state, both conformations existed, and the dual emission bands from the LE and ICT states were obtained by freezing at 77 K. From the PL spectra, three distinct emission bands were also observed from both the solutions containing **2B** and **3B**. Similarly to **1B**, these data indicate that two types of molecular distributions, such as the planar and twisted conformations, should be included in the ground state. This theoretical investigation also supports that the TICT emission should be obtained from the *o*-carborane-modified triphenylamines.

## 3. Conclusions

We accomplished the enhancing of AIE behaviors by introducing multiple *o*-carborane units into triphenylamine. According to the results from optical measurements, it was proposed that the second and third *o*-carborane units were responsible for improving emission efficiencies in the solid state. Indeed, intense emission bands were observed only from the solid samples in this study. Finally, clear AIE behaviors were obtained from **2B** and **3B**, indicating that *o*-carborane can play a significant role in enhancing the AIE property as well as AIE-inducible element-blocks. Our findings could be useful not only for designing AIE-active dyes, but also for developing environment-sensitive emissive materials.

## Figures and Tables

**Table 1:** Optical properties of **1**–**3B**.

Compound	*λ*_abs_ (nm) ^a^	*ε*_max_ (M^−1^ cm^−1^)	*λ*_em,rt_ (nm) ^b^	*λ*_em,rt_ (nm) ^c^	*Φ*_PL_^b,d^	*λ*_em,77K_ (nm) ^e^	*λ*_em,rt_ (n *E*_a_ + *ΔH E*_a_ + *ΔH* m) ^f^	*Φ*_PL_^d,f^	*λ*_em,77K_ (nm) ^f^	*E*_a_ (kJ/mol)	*ΔH* (kJ/mol)	*E*_a_ + *ΔH* (kJ/mol)
**1B**	314	23,300	374, 557	364, 567	<0.01	361, 424, 662	372, 635	0.04	372, 649	3.4	4.4	7.8
**2B**	331	28,900	362, 541	382, 694	<0.01	382, 460, 625	623	0.30	602	5.0	4.7	9.7
**3B**	321	45,300	358, 516	379, 677	<0.01	377, 452, 609	602	0.25	586	5.6	2.9	8.5

^a^ 1.0 × 10^−5^ M in octane at room temperature. ^b^ 1.0 × 10^−5^ M in octane at room temperature with the excitation light at *λ*_abs_. ^c^ 1.0 × 10^−5^ M in MTHF at room temperature with the excitation light at *λ*_abs_. ^d^ Determined as an absolute value with the integration sphere method. ^e^ 1.0 × 10^−5^ M in MTHF at 77 K with the excitation light at *λ*_abs_. ^f^ Measured in the solid state with the excitation light at *λ*_abs_.

## Supplementary Materials

Supplementary Materials are available online.

## References

[1] Chujo Y., Tanaka K. (2015). New polymeric materials based on element-blocks. Bull. Chem. Soc. Jpn..

[2] Mei J., Leung N.L.C., Kwok R.T.K., Lam J.W.Y., Tang B.Z. (2015). Aggregation-induced emission: Together we shine, united we soar!. Chem. Rev..

[3] Gon M., Tanaka K., Chujo Y. (2017). Recent progress in the development of advanced element-block materials. Polym. J..

[4] Tanaka K., Chujo Y. (2015). Recent progress of optical functional nanomaterials based on organoboron complexes with *β*-diketonate, ketoiminate and diiminate. NPG Asia Mater..

[5] Bregadze V.I. (1992). Dicarba-*closo*-dodecaboranes C_2_B_10_H_12_ and their derivatives. Chem. Rev..

[6] Scholz M., Hey-Hawkins E. (2011). Carbaboranes as pharmacophores: Properties, synthesis, and application strategies. Chem. Rev..

[7] Issa F., Kassiou M., Rendina L.M. (2011). Boron in drug discovery: Carboranes as unique pharmacophores in biologically active compounds. Chem. Rev..

[8] Núñez R., Romero I., Teixidor F., Viñas C. (2016). Inorganic dendrimers: Recent advances for catalysis, nanomaterials, and nanomedicine. Chem. Soc. Rev..

[9] Núñez R., Terrés M., Ferrer-Ugalde A., Biani F.F.D., Teixidor F. (2016). Electrochemistry of boron compounds. Chem. Rev..

[10] Grimes R.N. (2011). Carboranes.

[11] Li X., Yan H., Zhao Q. (2016). Carboranes as a tool to tune phosphorescence. Chem. Eur. J..

[12] Mukherjee S., Thilagar P. (2016). Boron clusters in luminescent materials. Chem. Commun..

[13] Böhling L., Brockhinke A., Kahlert J., Weber L., Harder R.A., Yufit D.S., Howard J.A.K., MacBride J.A.H., Fox M.A. (2016). Substituent effects on the fluorescence properties of *ortho*-carboranes: Unusual emission behaviour in *C*-(2′-pyridyl)-*ortho*-carboranes. Eur. J. Inorg. Chem..

[14] Weber L., Kahlert J., Brockhinke R., Böhling L., Halama J., Brockhinke A., Stammler H.-G., Neumann B., Nervi C., Harder R.A. (2013). *C,C*′-Bis(benzodiazaborolyl)dicarba-*closo*-dodecaboranes: Synthesis, structures, photophysics and electrochemistry. Dalton Trans..

[15] Kahlert J., Böhling L., Brockhinke A., Stammler H.-G., Neumann B., Rendina L.M., Low P.J., Weber L., Fox M.A. (2015). Syntheses and reductions of *C*-dimesitylboryl-1,2-dicarba-*closo*-dodecaboranes. Dalton Trans..

[16] Choi B.H., Lee J.H., Hwang H., Lee K.M., Park M.H. (2016). Novel dimeric *o*-carboranyl triarylborane: Intriguing ratiometric color-tunable sensor via aggregation-induced emission by fluoride anions. Organometallics.

[17] Eo M., Park M.H., Kim T., Do Y., Lee M.H. (2013). Polynorbornene copolymers with pendent *o*-carborane and carbazole groups: Novel side-chain donor-acceptor copolymers for turn-on sensing of nucleophilic anions. Polymer.

[18] Kim T., Kim H., Lee K.M., Lee Y.S., Lee M.H. (2013). Phosphorescence color tuning of cyclometalated iridium complexes by *o*-carborane substitution. Inorg. Chem..

[19] Peterson J.J., Davis A.R., Were M., Coughlin E.B., Carter K.R. (2011). Carborane-containing poly(fluorene): Response to solvent vapors and amines. ACS Appl. Mater. Interfaces.

[20] Tu D., Leong P., Li Z., Hu R., Shi C., Zhang K.Y., Yan H., Zhao Q. (2016). A carborane-triggered metastable charge transfer state leading to spontaneous recovery of mechanochromic luminescence. Chem. Commun..

[21] Zhang W., Luo Y., Xu Y., Tian L., Li M., He R., Shen W. (2015). The electronic structures and photophysical properties of platinum complexes with C^N^N ligands: The influence of the carborane substituent. Dalton Trans..

[22] Zhu L., Tang X., Yu Q., Lv W., Yan H., Zhao Q., Huang W. (2015). Tuning the optical properties of 2-thienylpyridyl iridium complexes through carboranes and anions. Chem. Eur. J..

[23] Cho Y.-J., Kim S.-Y., Cho M., Han W.-S., Son H.-J., Cho D.W., Kang S.O. (2016). Aggregation-induced emission of diarylamino-π-carborane triads: Effects of charge transfer and π-conjugation. Phys. Chem. Chem. Phys..

[24] Kim S.-Y., Cho Y.-J., Jin G.F., Han W.-S., Son H.-J., Cho D.W., Kang S.O. (2015). Intriguing emission properties of triphenylamine-carborane systems. Phys. Chem. Chem. Phys..

[25] Wang Z., Jiang P., Wang T., Moxey G.J., Cifuentes M.P., Zhang C., Humphrey M.G. (2016). Blue-shifted emission and enhanced quantum efficiency via p-bridge elongation in carbazole–carborane dyads. Phys. Chem. Chem. Phys..

[26] Kwon S., Wee K.-R., Cho Y.-J., Kang S.O. (2014). Carborane dyads for photoinduced electron transfer: Photophysical studies on carbazole and phenyl-*o*-carborane molecular assemblies. Chem. Eur. J..

[27] Naito H., Nishino K., Morisaki Y., Tanaka K., Chujo Y. (2017). Highly-efficient solid-state emissions of the anthracene-*o*-carborane dyads with various substituents and their thermochromic luminescent properties. J. Mater. Chem. C.

[28] Nishino K., Yamamoto H., Tanaka K., Chujo Y. (2016). Development of solid-state emissive materials based on multi-functional *o*-carborane-pyrene dyads. Org. Lett..

[29] Naito H., Nishino K., Morisaki Y., Tanaka K., Chujo Y. (2017). Luminescence color tuning of stable luminescent solid materials from blue to NIR based on bis-*o*-carborane-substituted oligoacenes. Chem. Asian J..

[30] Furue R., Nishimoto T., Park I.S., Lee J., Yasuda T. (2016). Aggregation-induced delayed fluorescence based on donor/acceptor-tethered Janus carborane triads: Unique photophysical properties of nondoped OLEDs. Angew. Chem. Int. Ed..

[31] Wee K.-R., Cho Y.-J., Jeong S., Kwon S., Lee J.-D., Suh I.-H., Kang S.O. (2012). Carborane-based optoelectronically active organic molecules: Wide band gap host materials for blue phosphorescence. J. Am. Chem. Soc..

[32] Inagi S., Hosoi K., Kubo T., Shida N., Fuchigami T. (2013). *o*-Carborane-triphenylamine dyad: Studies on its acceptor-donor behavior toward dual redox mediator. Electrochemistry.

[33] Son M.R., Cho Y.-J., Kim S.-Y., Son H.-J., Cho D.W., Kang S.O. (2017). Direct observation of the photoinduced electron transfer processes of bis(4-arylphenylamino benzo)-*ortho*-carborane using transient absorption spectroscopic measurements. Phys. Chem. Chem. Phys..

[34] Tu D., Leong P., Guo S., Yan H., Lu C., Zhao Q. (2017). Highly emissive organic single-molecule white emitters by engineering *o*-carborane-based luminophores. Angew. Chem. Int. Ed..

[35] Chen Y., Guo J., Wu X., Jia D., Tong F. (2018). Color-tuning aggregation-induced emission of *o*-carborane-bis(1,3,5-triaryl-2-pyrazoline) triads: Preparation and investigation of the photophysics. Dyes Pigment..

[36] Ferrer-Ugalde A., Cabrera-González J., Juárez-Pérez E.J., Teixidor F., Pérez-Inestrosa E., Montenegro J.M., Sillanpää R., Haukka M., Núñez R. (2017). Carborane-stilbene dyads: The influence of substituents and cluster isomers on photoluminescence properties. Dalton Trans..

[37] Wang Z., Wang T., Zhang C., Humphrey M.G. (2017). Efficient crystallization-induced emission in fluorenyl-tethered carboranes. Phys. Chem. Chem. Phys..

[38] Li X., Yin Y., Yan H., Lu C. (2017). Aggregation-induced emission characteristics of *o*-carborane-functionalized tetraphenylethylene luminogens: The influence of carborane cages on photoluminescence. Chem. Asian J..

[39] Tanaka K., Nishino K., Ito S., Yamane H., Suenaga K., Hashimoto K., Chujo Y. (2017). Development of the Solid-State Emissive *o*-Carborane and Theoretical Investigation for Mechanism of Aggregation-Induced Emission Behaviors of Organoboron “Element-Blocks”. Faraday Discuss..

[40] Nishino K., Hashimoto K., Tanaka K., Morisaki Y., Chujo Y. (2016). Synthesis and properties of highly-rigid conjugation system based on bi(benzo[*b*]thiophene)-fused *o*-carborane. Tetrahedron Lett..

[41] Nishino K., Morisaki Y., Tanaka K., Chujo Y. (2017). Electron-donating abilities and luminescent properties of tolane-substituted *nido*-carboranes. New J. Chem..

[42] Nishino K., Yamamoto H., Tanaka K., Chujo Y. (2017). Solid-state themochromic luminescence via twisted intramolecular charge transfer and excimer formation of the carborane-pyrene dyad with an ethynyl spacer. Asian J. Org. Chem..

[43] You D.K., Lee J.H., Choi B.H., Hwang H., Lee M.H., Lee K.M., Par M.H. (2017). Effects of multi-carborane substitution on the photophysical and electron-accepting properties of *o*-carboranylbenzene compounds. Eur. J. Inorg. Chem..

[44] Zhu L., Lv W., Liu S., Yan H., Zhao Q., Huang W. (2013). Carborane enhanced two-photon absorption of tribranched fluorophores for fluorescence microscopy imaging. Chem. Commun..

[45] Nicoud J.-F., Bolze F., Sun X.-H., Hayek A., Baldeck P. (2011). Boron-containing two-photon-absorbing chromophores. 3. One- and two-photon photophysical properties of *p*-carborane-containing fluorescent bioprobes. Inorg. Chem..

[46] Uebe M., Ito A., Kameoka Y., Sato T., Tanaka K. (2015). Fluorescence enhancement of non-fluorescent triphenylamine: A recipe to utilize carborane cluster substituents. Chem. Phys. Lett..

[47] Kameoka Y., Uebe M., Ito A., Sato T., Tanaka K. (2014). Fluorescent triphenylamine derivative: Theoretical design based on reduced vibronic coupling. Chem. Phys. Lett..

[48] Rajavelu K., Rajakumar P., Sudip M., Kothandaraman R. (2016). Synthesis, photophysical, electrochemical, and DSSC application of novel donor-acceptor triazole bridged dendrimers with a triphenylamine core and benzoheterazole as a surface unit. New J. Chem..

[49] Tydlitát J., Achelle S., Rodríguez-López J., Pytela O., Mikýsek T., Cabon N., Guen F.R., Miklík D., Růžičková Z., Bureš F. (2017). Photophysical properties of acid-responsive triphenylamine derivatives bearing pyridine fragments: Towards white light emission. Dyes Pigment..

[50] Cvejn D., Michail E., Seintis K., Klikar M., Pytela O., Mikysek T., Almonasy N., Ludwig M., Giannetas V., Fakis M. (2016). Solvent and branching effect on the two-photon absorption properties of push-pull triphenylamine derivatives. RSC Adv..

[51] Hrobárik P., Hrobáriková V., Sigmundová I., Zahradník P., Fakis M., Polyzos I., Persephonis P. (2011). Benzothiazoles with tunable electron-withdrawing strength and reverse polarity: A route to triphenylamine-based chromophores with enhanced two-photon absorption. J. Org. Chem..

[52] Romain M., Tondelier D., Jeannin O., Geffroy B., Rault-Berthelot J., Poriel C. (2015). Properties modulation of organic semi-conductors based on a donor-spiro-acceptor (D-spiro-A) molecular design: New host materials for efficient sky-blue PhOLEDs. J. Mater. Chem. C.

[53] Naito H., Nishino K., Morisaki Y., Tanaka K., Chujo Y. (2017). Solid-state emission of the anthracene-*o*-carborane dyad via twisted-intramolecular charge transfer in the crystalline state. Angew. Chem. Int. Ed..

[54] Matsumoto T., Takamine H., Tanaka K., Chujo Y. (2017). Design of bond-cleavage-induced intramolecular charge transfer emission with dibenzoboroles and their application to ratiometric sensors for discriminating chain lengths of alkanes. Mater. Chem. Front..

[55] Weber L., Kahlert J., Brockhinke R., Böhling L., Brockhinke A., Stammler J.-G., Neumann B., Harder R.A., Fox M.A. (2012). Luminescence properties of *C*-diazaborolyl-ortho-carboranes as donor-acceptor systems. Chem. Eur. J..

