# Supplementary material for: Enhancement of Aggregation-Induced Emission by Introducing Multiple o-Carborane Substitutions into Triphenylamine
Source: Molecules. 2017 Nov 19;22(11):2009. doi: 10.3390/molecules22112009 (PMC6150215; doi:10.3390/molecules22112009)
Supplement: Supplementary file 1 [file molecules-22-02009-s001.pdf]

Supplementary Materials: Enhancement of Aggregation-Induced Emission Property by Introducing Multiple Substitutions of *o*-Carborane into Triphenylamine

Kenta Nishino, Kyoya Uemura, Masayuki Gon, Kazuo Tanaka*, and Yoshiki Chujo*

Department of Polymer Chemistry, Graduate School of Engineering, Kyoto University, Katsura, Nishikyo-ku, Kyoto 615-8510, Japan

E-mail: tanaka@poly.synchem.kyoto-u.ac.jp; chujo@poly.synchem.kyoto-u.ac.jp

Experimental section

General

^{1}H, ^{13}C, and ^{11}B NMR spectra were recorded on a JEOL JNM-EX400 instrument at 400, 100, and 128 MHz, respectively. The ^{1}H and ^{13}C chemical shift values were expressed relative to Me_4Si as an internal standard. The ^{11}B chemical shift values were expressed relative to $BF_3{\cdot}Et_2O$ as an external standard. High-resolution mass spectra (HRMS) were obtained on a Thermo Fisher Scientific EXACTIVE spectrometer for atmospheric pressure chemical ionization (APCI). Analytical thin-layer chromatography (TLC) was performed with silica gel 60 Merck F254 plates. Column chromatography was performed with Wakogel C-300 silica gel. UV–vis absorption spectra were obtained on a SHIMADZU UV3600 spectrophotometer. Photoluminescence (PL) spectra were obtained on a Horiba FluoroMax-4 luminescence spectrometer. Absolute PL quantum efficiencies (Φ_{PL}) were determined using a Horiba FL-3018 Integrating Sphere. **2a** and **3a** were prepared according to the previous literature.[1]

Synthetic procedures

1B. Decaborane (291 mg, 2.39 mmol) was dissolved in CH_3CN (2 mL) at room temperature under Ar atmosphere. The mixture was stirred for 1 h at 50 °C, and then, dry toluene (20 mL), $AgNO_3$ (25.8 mg, 0.152 mmol) and 4-ethynyltriphenylamine (533 mg, 1.98 mmol) were added. The mixture was refluxed for 2 d. After cooling to room temperature, the solvent was evaporated, and the residue was subjected to silica gel column chromatography with hexane/CH_2Cl_2 (v/v = 9/1) as an eluent. Recrystallization from chloroform and methanol (good and poor solvent, respectively) to give **1B** as a yellow solid (54.4 mg, 6%).^{1}H NMR (400 MHz, $CDCl_3$): δ (ppm) 7.29 (s, 6H, Ar–*H*), 7.10 (1H, s, Ar–*H*), 6.90 (d, 2H, *J* = 9.0, Ar–*H*), 3.99 (s, 1H, carborane_C–*H*), 3.8–1.80 (br, 10H, B–*H*). ^{13}C NMR (100 MHz, $CDCl_3$): δ (ppm) 149.4, 146.8, 129.5, 128.7, 125.5, 125.5, 124.2, 120.9, 61.0, 29.7. ^{11}B NMR (128 MHz, $CDCl_3$): δ (ppm) –1.5, –2.6, –4.5, –5.4, –8.7, –9.9, –11.3, –12.2, –13.4. HRMS (APCI^{+}): Calcd. for $C_{20}H_{26}B_{10}N$ $(M+H)^{+}$ m/z 390.2990, found m/z 390.2990.

2B. Decaborane (632 mg, 5.18 mmol) was dissolved in CH_3CN (2.3 mL) at room temperature under Ar atmosphere. The mixture was stirred for 2 h at 50 °C, and then, dry toluene (22.5 mL), $AgNO_3$ (30.6 mg, 0.180 mmol) and the ethynyl compound[1] (606 mg, 2.25 mmol) were added. The mixture was refluxed for 7 d. After cooling to room temperature, the solvent was evaporated, and the residue was subjected to silica gel column chromatography with hexane/CH_2Cl_2 (v/v = 4/1) as an eluent. Recrystallization from chloroform and methanol (good and poor solvent, respectively) to give **2B** as a yellow solid (0.0869 g, 10%). ^1H NMR (400 MHz, CD_2Cl_2): δ (ppm) 7.30–7.38 (m, 6H, Ar–*H*), 7.17 (t, 1H, *J* = 7.4 Hz, Ar–*H*), 7.08 (d, 2H, *J* = 7.3 Hz, Ar–*H*), 6.96 (d, 4H, *J* = 9.0 Hz, Ar–*H*), 4.00 (s, 2H, carborane_C–*H*), 3.2–1.6 (br, 20H, B–*H*). ^{13}C NMR (100 MHz, CD_2Cl_2): δ (ppm) 149.0, 146.5, 130.2, 129.3, 127.6, 126.6, 125.6, 123.2, 77.4, 61.5. ^{11}B NMR (128 MHz, CD_2Cl_2): δ (ppm) –1.7, –2.8, –4.9, –8.6, –10.0, –10.7, –11.4, –12.1, –13.4. HRMS(APCI$^+$) $C_{22}H_{36}B_{20}N_1$ $[M+H]^+$: calcd. 534.4773, found 534.4758.

3B. Decaborane (527 mg, 4.31 mmol) was dissolved in CH_3CN (2.5 mL) at room temperature under Ar atmosphere. The mixture was stirred for 2 h at 50 °C, and then, dry toluene (31.3 mL), $AgNO_3$ (34.0 mg, 0.200 mmol) and the ethynyl compound[1] (397 mg, 1.25 mmol) were added. The mixture was refluxed for 7 d. After cooling to room temperature, the solvent was evaporated, and the residue was subjected to silica gel column chromatography with hexane/CH_2Cl_2 (v/v = 4/1) as an eluent. Recrystallization from chloroform and methanol (good and poor solvent, respectively) to give **3B** as a yellow solid (44.4 mg, 5%). ^1H NMR (400 MHz, $CDCl_3$): δ (ppm) 7.38 (d, 6H, *J* = 7.3 Hz, Ar–*H*), 6.96 (d, 6H, *J* = 7.6 Hz, Ar–*H*), 3.89 (s, 3H, carborane_C–*H*), 3.6–1.6 (br, 30H, B–*H*). ^{13}C NMR (100 MHz, $CDCl_3$): δ (ppm) 147.7, 129.2, 128.7, 124.0, 77.1, 60.6. ^{11}B NMR (128 MHz, $CDCl_3$): δ (ppm) –1.3, –2.4, –4.6, –8.4, –9.6, –10.1, –11.3, –12.0. HRMS(APCI$^-$) $C_{24}H_{45}B_{30}N_1Cl_1[M+Cl]^-$: calcd. 712.6081, found 712.6067.

References

1. Rajavelu, K.; Rajakumar, P.; Sudip, M.; Kothandaraman, R. *New J. Chem.* **2016**, *40*, 10246.

Figures and Table

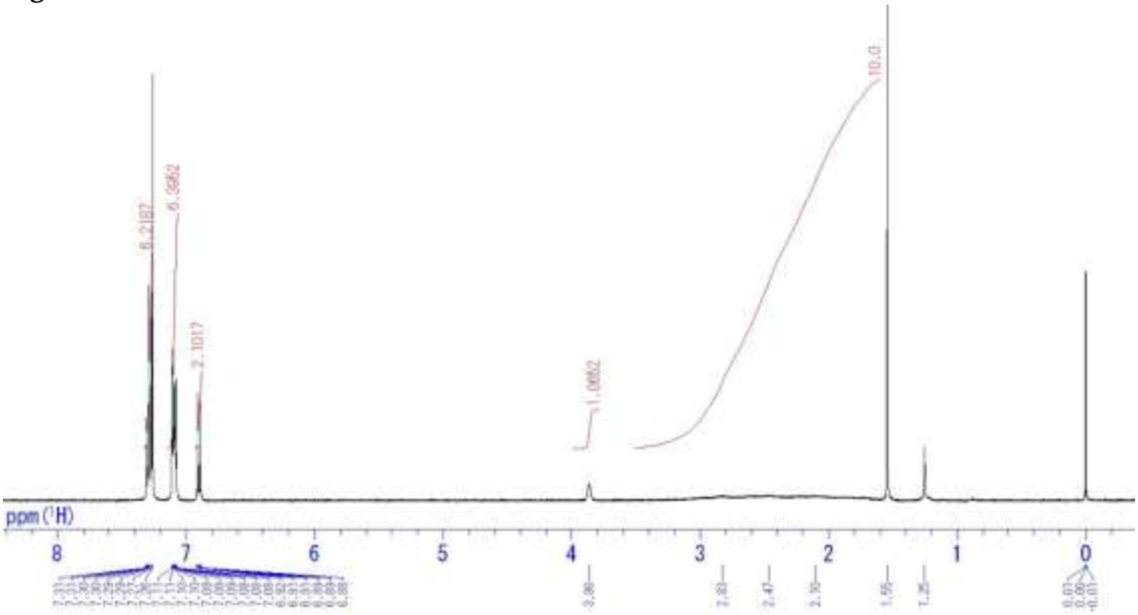

Chart S1. ^{1}H NMR spectrum of **1B** in $CDCl_3$ at room temperature.

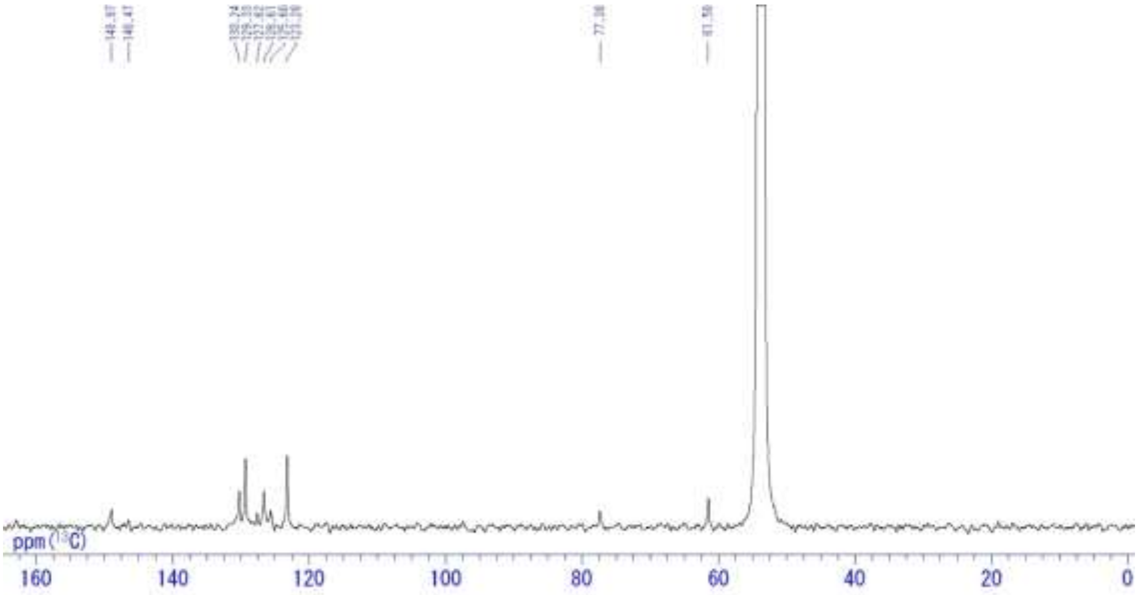

Chart S2. ^{13}C NMR spectrum of **1B** in $CDCl_3$ at room temperature.

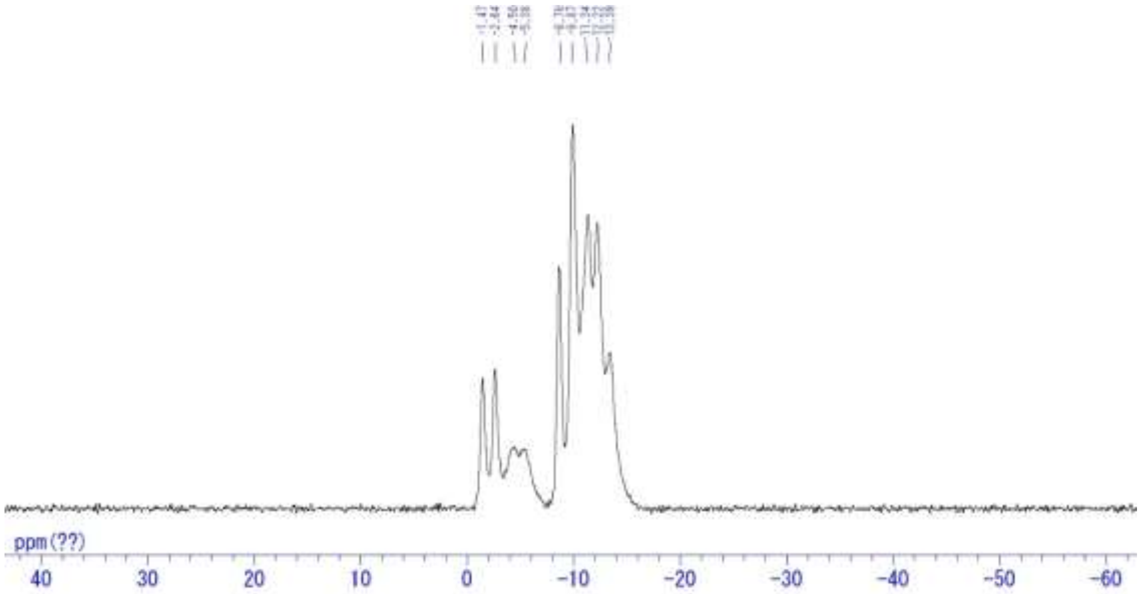

Chart S3. ^{11}B NMR spectrum of **1B** in $CDCl_3$ at room temperature.

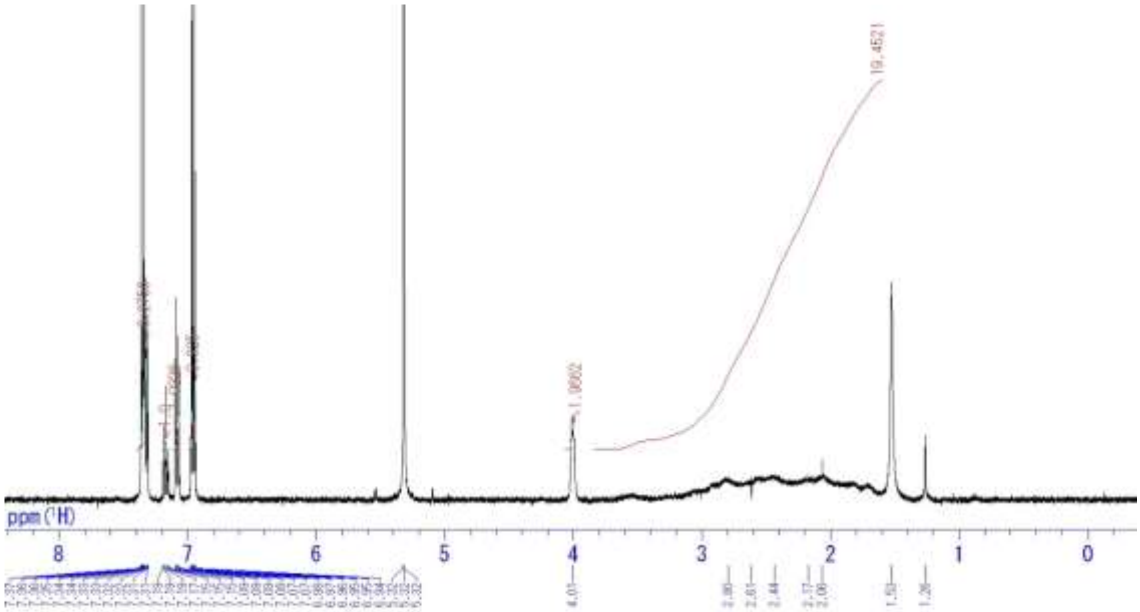

Chart S4. ^{1}H NMR spectrum of **2B** in CD_2Cl_2 at room temperature.

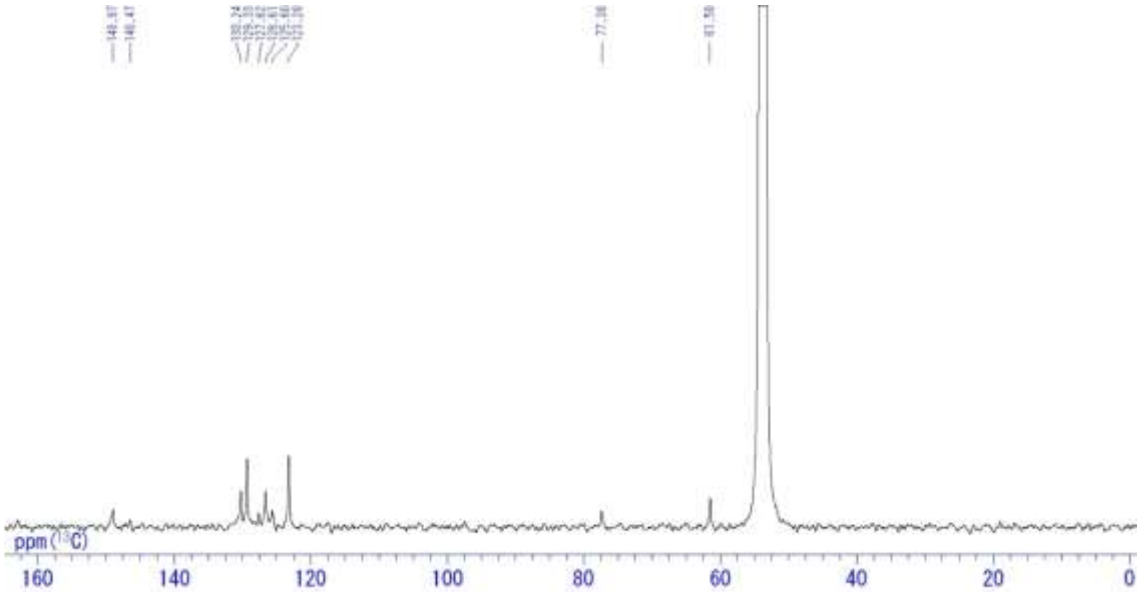

Chart S5. ^{13}C NMR spectrum of **2B** in CD_2Cl_2 at room temperature.

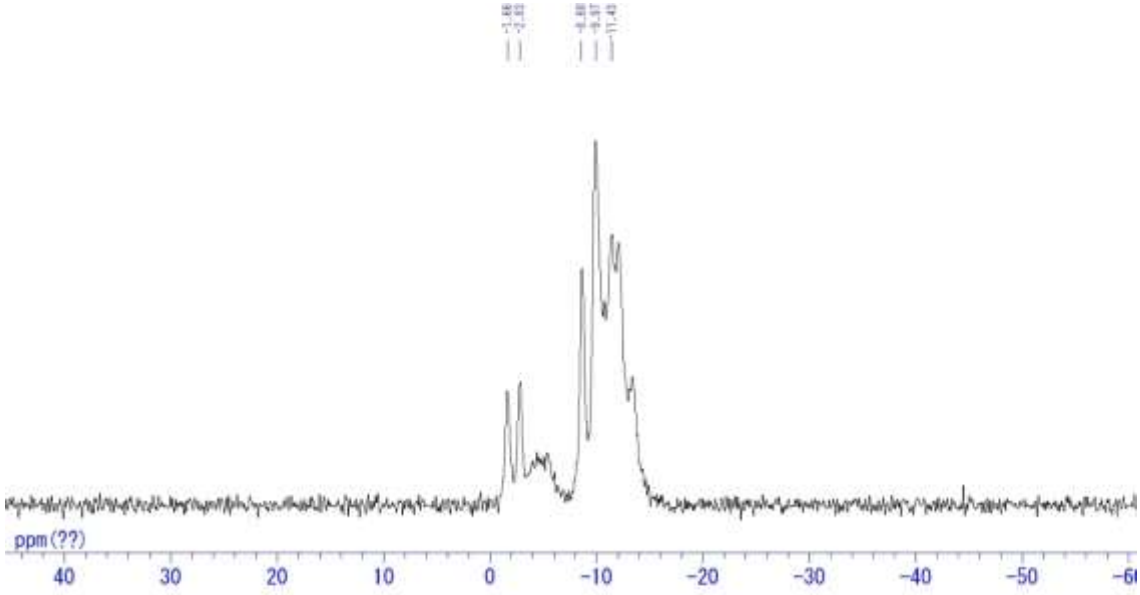

Chart S6. ^{11}B NMR spectrum of **2B** in CD_2Cl_2 at room temperature.

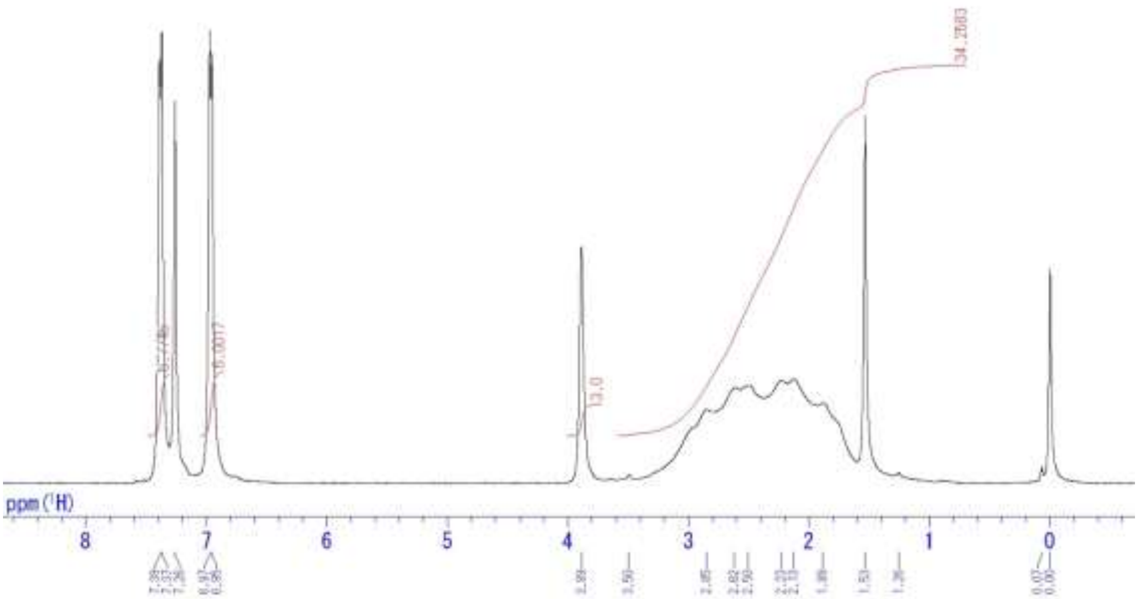

Chart S7. ^{1}H NMR spectrum of **3B** in $CDCl_3$ at room temperature.

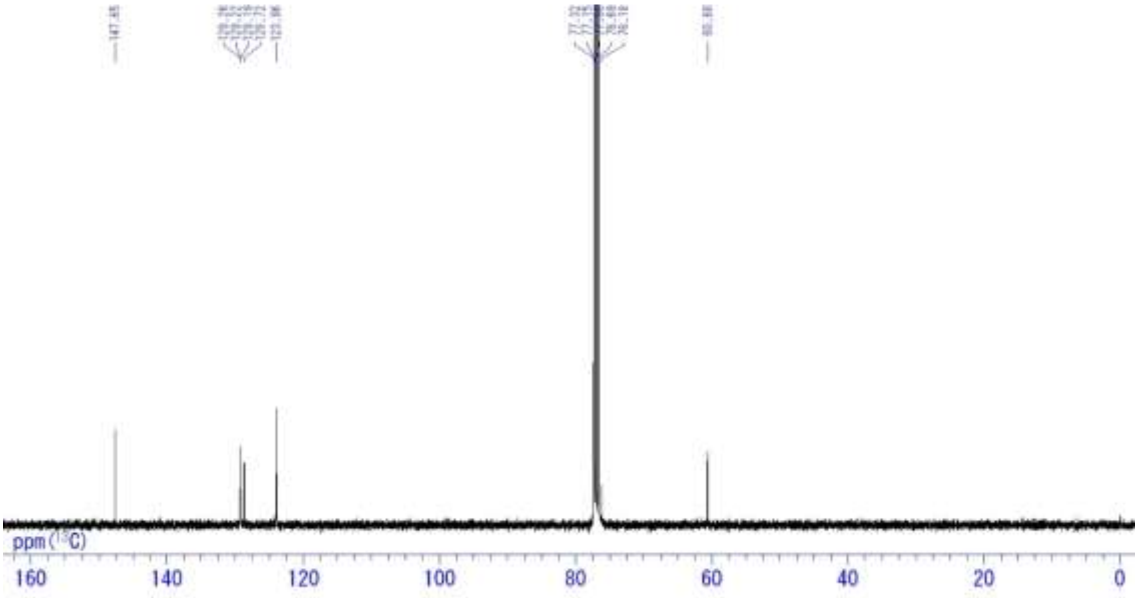

Chart S8. ^{13}C NMR spectrum of **3B** in $CDCl_3$ at room temperature.

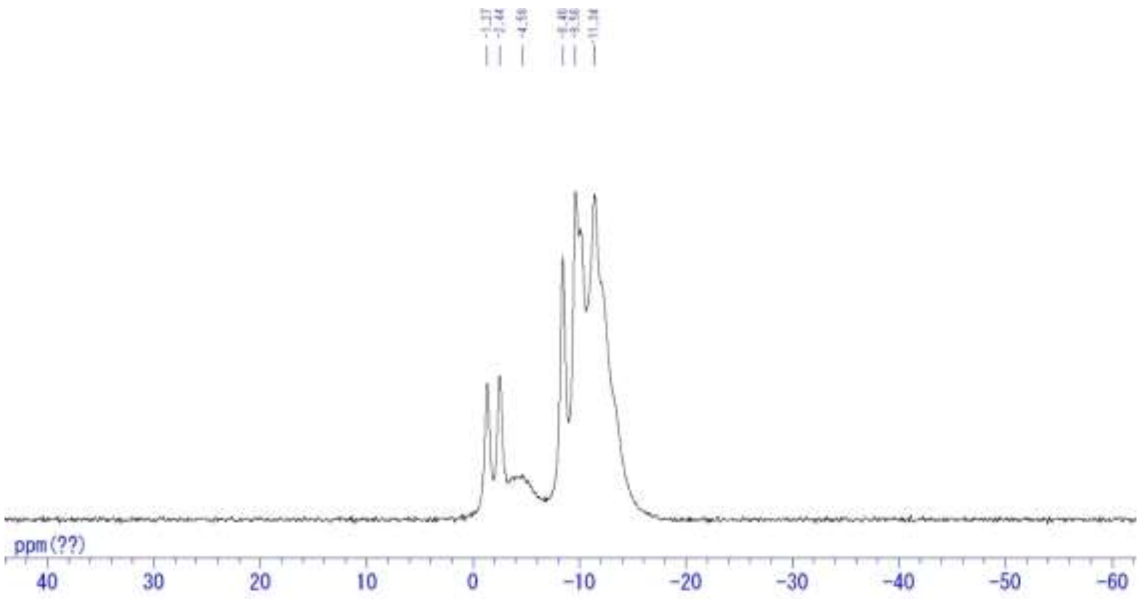

Chart S9. ^{11}B NMR spectrum of **3B** in $CDCl_3$ at room temperature.

Table S1. Optical properties of triphenylamine

Parameter	Value
λ_{abs} (nm)[a]	300
ε_{max} ($M^{-1}cm^{-1}$)[a]	27,700
$\lambda_{em,rt}$ (nm)[b]	358
$\lambda_{em,77K}$ (nm)[c]	353, 406, 428

[a] 1.0×10^{-5} M in THF at room temperature. [b] 1.0×10^{-5} M in THF at room temperature with the excitation light at 300 nm. [c] 1.0×10^{-5} M in MTHF at room temperature with the excitation light at 300 nm.

Lippert–Mataga Plots

Lippert–Mataga plots were conducted with the following formula.[3]

$$\tilde{\nu}_{\mathrm{abs}} - \tilde{\nu}_{\mathrm{PL}} = \frac{2\Delta f}{4\pi\varepsilon_0 hca^3}\Delta\mu^2 + \mathrm{const.}$$

where ε_0 is the permittivity of vacuum h equals Planck's constant, c equals the velocity of light in a vacuum, a is the Onsager cavity radius, n and ε are the refractive index and the dielectric constant of the solvent, $\tilde{\nu}_{\mathrm{abs}}$ and $\tilde{\nu}_{\mathrm{PL}}$ are the absorption and photoluminescence wavenumber, $\Delta\mu$ is the difference in dipole moments during the transition, and Δf is the orientation polarizability defined as below, respectively.

$$\Delta f = \frac{\varepsilon - 1}{2\varepsilon + 1} - \frac{n^2 - 1}{2n^2 + 1}$$

Hence the slope derived from a plot of Stokes-shifts ($\tilde{\nu}_{\mathrm{abs}} - \tilde{\nu}_{\mathrm{PL}}$) as a function of Δf can be expressed as

$$\mathrm{slope} = \frac{2}{4\pi\varepsilon_0 hca^3}\Delta\mu^2$$

Table S2. Optical properties of the triphenylamine derivatives in various solvents

	**1B**		**2B**		**3B**	
	λ_{em} (nm)	τ (ns)	λ_{em} (nm)	τ (ns)	λ_{em} (nm)	τ (ns)
Hexane	380, 570	2.02, 4.81	381, 545	1.91, 4.06	383, 525	2.11, 3.77
$CHCl_3$	390	1.63	385, 660	1.90, 3.88	384, 619	1.98, 4.66
THF	385, 450	–	385	–	385, 680	–
CH_2Cl_2	390	2.32	386	2.09	382, 670	2.09, 4.32
MeCN	390	2.17	383	2.10	391	2.13

Table S3. Results of Lippert–Mataga plots[a]

Compound	Slope / cm^{-1}	$\Delta\mu$ / Deby[b]
**2B**	10,328	14.0
**3B**	9,644	13.5

[a]Due to large ICT character of **1B**, the significant values were not detectable. [b]Effective radii of the Onsager cavity were estimated from the length between the nitrogen atom in the TPA moiety and the aryl-connected carbon atom in the *o*-carborane unit.

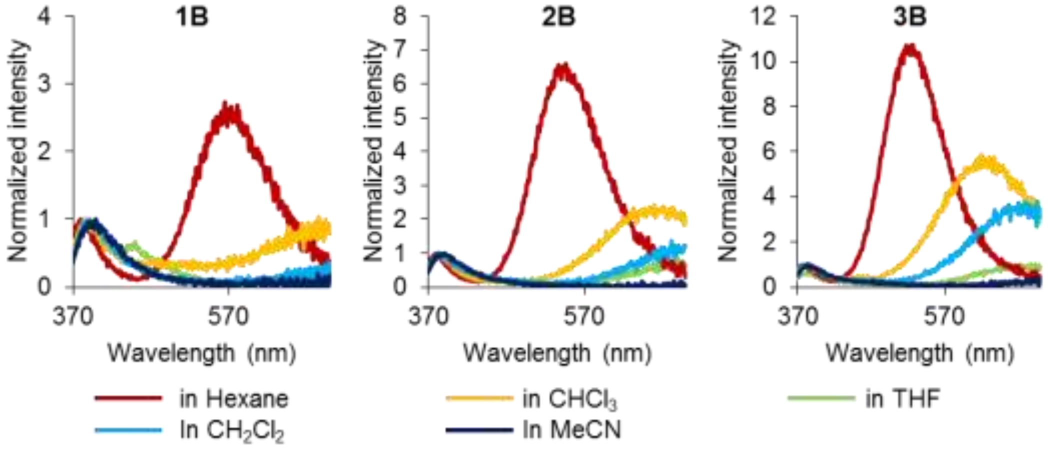

Figure S1. Solvent dependent PL spectra of **1–3B**.

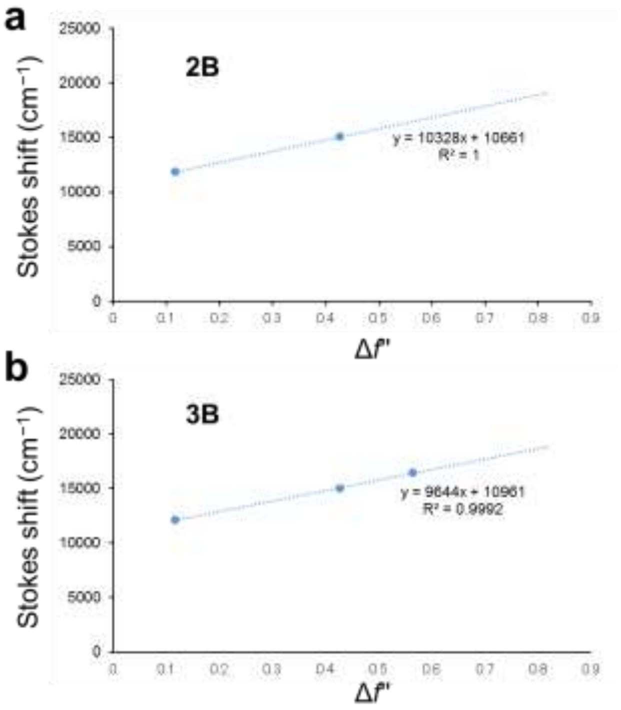

Figure S2. Lippert–Mataga plots with **2B** and **3B**.

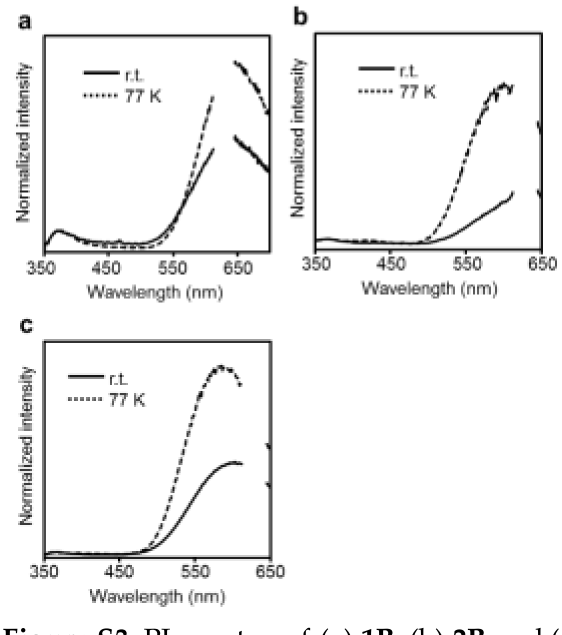

Figure S3. PL spectra of (a) **1B**, (b) **2B** and (c) **3B** in the solid state at 77 K.

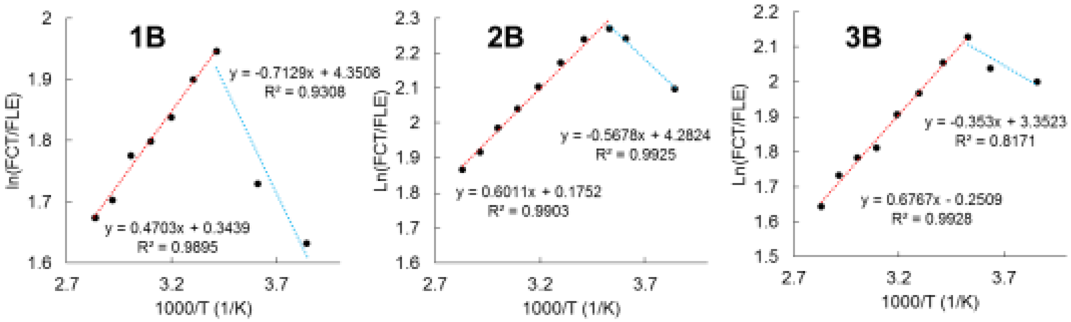

Figure S4. Stevens–Ban plots with **1–3B**.

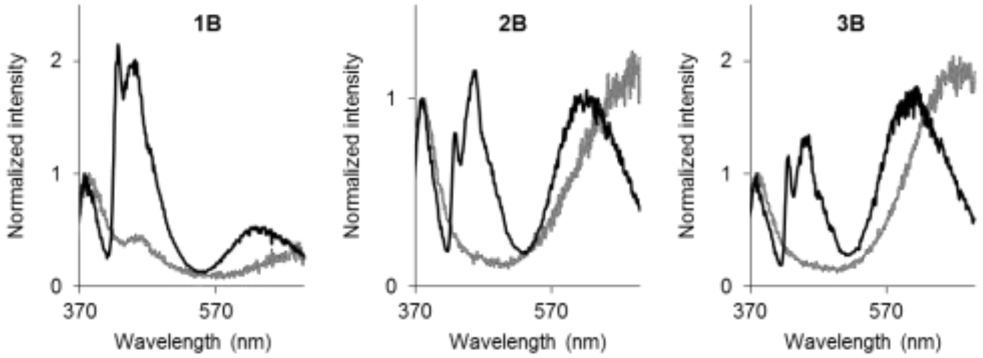

Figure S5. Normalized PL spectra of **1–3B** in MTHF (1.0×10^{-5} M) at r.t. (gray line) and 77 K (black line).

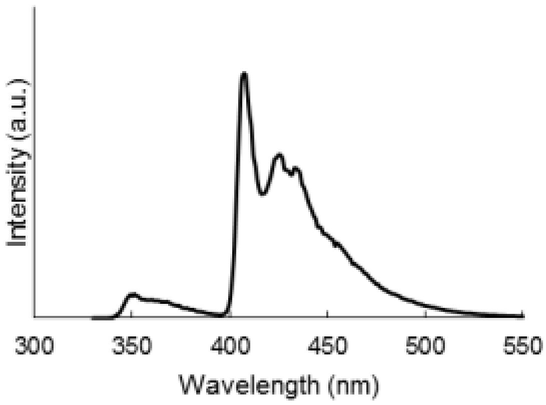

Figure S6. PL spectrum of triphenylamine in MTHF at 77 K.
